# STVF: Spatial-Temporal Variational Filtering for Localization in Underwater Acoustic Sensor Networks

**DOI:** 10.3390/s18072078

**Published:** 2018-06-28

**Authors:** Keyong Hu, Zhongwei Sun, Hanjiang Luo, Wei Zhou, Zhongwen Guo

**Affiliations:** 1 School of Information and Control Engineering, Qingdao University of Technology, Qingdao 266520, China; hukeyongouc@163.com (K.H.); zhouwei@qut.edu.cn (W.Z.); 2 School of Computer Science and Engineering, Shandong University of Science and Technology, Qingdao 266590, China; luo.wireless@gmail.com; 3 Department of Computer Science and Technology, Ocean University of China, Qingdao 266100, China; guozhw@163.com

**Keywords:** underwater acoustic sensor networks, variational filtering, spatial correlation, temporal dependency, iterative localization

## Abstract

Localization is one of the critical services in Underwater Acoustic Sensor Networks (UASNs). Due to harsh underwater environments, the nodes often move with currents continuously. Consequently, the acoustic signals usually propagate with varying speeds in non-straight lines and the noise levels change frequently with the motion of the nodes. These limitations pose huge challenges for localization in UASNs. In this paper, we propose a novel localization method based on a variational filtering technique, in which the spatial correlation and temporal dependency information are utilized to improve localization performance. In the method, a state evolution model is employed to characterize the mobility pattern of the nodes and capture the uncertainty of the location transition. Then, a measurement model is used to reflect the relation between the measurements and the locations considering the dynamics of the acoustic speed and range noise. After that, a variational filtering scheme is adopted to determine the nodes’ locations, which consists of two phases: variational prediction and update. In the former phase, the coarse estimation of each node’ location is computed based on its previous location; in the latter phase, the coarse location is optimized by incorporating the measurements from the reference nodes as precisely as possible. At last, an iterative localization scheme is applied, in which a node labels itself as a reference node if the confidence of its location estimation is higher than the predefined threshold. We conducted extensive simulations under different parameter settings, and the results indicate that the proposed method has better localization accuracy compared to a typical SLMP algorithm while maintaining relatively high localization coverage. Moreover, spatial–temporal variational filtering (STVF) is more robust to the change of the parameter settings compared to SLMP.

## 1. Introduction

Underwater Acoustic Sensor Networks (UASNs) have attracted much attention due to its application in many fields such as environmental monitoring, military defense, disaster prevention and ocean resource exploration [1,2,3]. In UASNs, underwater nodes measure water phenomena’s values with equipped sensors, communicate with each other by acoustic signals and collaborate to send the sensed data to the sink node. In this process, nodes’ locations need to be aware to support topology control, routing decision, subsequent data processing, etc. [4]. Therefore, localization is one of the most essential and fundamental services in UASNs.

Although there has been a lot of research for localization in terrestrial wireless sensor networks, they can’t be directly applied to UASNs due to the following reasons: (1) the acoustic communication has long propagation delay, and the acoustic signals often propagate with varying speeds in non-straight lines due to the inhomogeneity in terms of temperature, salinity and pressure; and (2) underwater nodes move with tides and currents continuously, resulting in frequent changes of network topology. These unique characteristics of underwater environment pose huge challenges for localization in UASNs.

Recently, many localization methods for UASNs have been widely explored [5,6]. Most of them assume that there are sufficient beacon nodes in the localization process. The unknown nodes perform localization using the spatial correlation between nodes (e.g., the distance estimations to neighbor beacon nodes). The localization performance is usually subject to the ranging accuracy and the algorithm efficiency. In many cases, UASNs are sparse since a limited number of nodes are deployed in a wide area. Moreover, the beacon nodes account for a small proportion in consideration of cost and efficiency. It is often the case that the unknown nodes can’t be localized due to the lack of sufficient beacon nodes, which results in low localization coverage.

In addition, ambient noise is an important factor that impacts localization performance, especially for range-based localization methods. Most existing works model the range noise as a Gaussian variable with fixed mean and variance, which can’t reflect the actual situations of harsh underwater surroundings. Generally, the range noises have two features: (1) the noises depend on the propagation path of the acoustic signal and change with the motion of underwater nodes [3]; (2) the range error usually increases with the distance between two nodes, which implies that the noise is closely related to the distance.

The characteristic of continuous mobility of underwater nodes has its own disadvantage and advantage. On one hand, a localized node will become unlocalized when beacon nodes move out of its communication range; on the other hand, the continuous movement of each node implies that the locations in its trajectory are dependent temporally. For example, the current location of one node is closely related to its locations at previous and next time instants. However, this temporal dependency among locations is neglected in most existing works.

**Figure 1:**
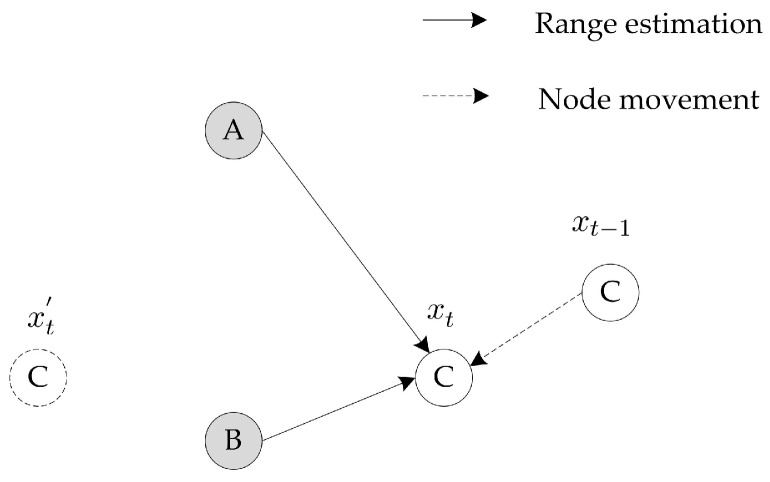
Localization with two beacon nodes.

In fact, localization based on spatial correlation in UASNs, especially with insufficient beacon nodes in sparse deployments, can be improved significantly using temporal dependency information. As illustrated in Figure 1, the location estimation of node C at time t−1 is denoted as $x_{t-1}$. It moves with currents and becomes neighbors of beacon nodes A and B at time *t*. Localization with two beacons will calculate two candidate locations $x_{t}$ and $x_{t}^{\prime }$. It is obvious that $x_{t}$ is the correct estimation under the assumption of low current velocity and short localization period.

To fuse spatial correlation and temporal dependency effectively, the approach commonly adopted is Bayesian filtering, which has been widely used in target tracking [7,8,9,10,11]. It attempts to obtain the posterior probability distribution of the state based on the available measurements. Since the measurements such as distance estimations are nonlinear functions of the state, the analytic solutions of the state estimation are intractable. Hence, the approximation approaches are needed. The Monte Carlo sampling methods such as Particle Filtering (PF) approximate a posterior probability distribution by use of a set of weighted samples. This method is not suitable for resource-constrained UASNs due to huge computation and storage costs. Alternatively, variational inference aims to find some decomposable distribution over the state to approximate a posterior distribution. It can accommodate an arbitrary measurement model and the convergence can be guaranteed. Aiming at the above challenges in UASN localization, we propose a spatial–temporal variational filtering algorithm, which is named STVF. It provides real-time localization in a distributed manner. First, we use a general state transition model to characterize the mobility pattern of the nodes. In virtue of this model, each node can predict its coarse location based on its previous location. This coarse location is then updated according to Bayes rule. Specifically, the posterior probability distribution of the node location can be obtained by taking the predicted location as a priori information and incorporating the spatial measurements as the likelihood information. Considering that the spatial measurements are the nonlinear function of the node location, we adopt an optimization method based on variational inference, in which the state variables related to the node location are optimized in turn. After the convergence value of the node location is obtained, we calculate the confidence value of the nodes, label the nodes with high confidence as reference nodes and localize the rest nodes iteratively. Therefore, the localization accuracy and coverage are both guaranteed in the STVF algorithm.

The rest of this paper is organized as follows: in Section 2, existing related works on UASN localization are briefly reviewed. Then, in Section 3, the network model is given and the UASN localization problem is formulated. Section 4 presents the design details of the STVF algorithm, in which the state evolution model and the measurement model are studied to support subsequent variational prediction and update phases. In Section 5, performances of the STVF algorithm are evaluated by simulations and are compared with the similar SLMP algorithm. Finally, we conclude the paper in Section 6.

## 2. Related Work

Existing localization schemes for UASN can be divided into two categories: range-free and range-based. While the range-free scheme is rarely adopted in realistic environments due to its poor localization accuracy, the range-based scheme has been paid more attention in recent literature. Ranging methods include Time of Arrival (TOA), Time Difference of Arrival (TDOA) , Angle of Arrival (AOA) and Received Signal Strength Indicator (RSSI) [12]. RSSI relies on an accurate signal attenuation model, which is hard to obtain in the time-varying acoustic environment. AOA necessitates the nodes to be equipped with directional antennas, resulting in additional costs. TDOA needs complex computation and thus consumes more energy [13]. In contrast, TOA is preferred in UASN localization since a long propagation delay provides high time resolution for distance estimation.

The TOA method gets the difference of message sending and receiving time, and then calculates distance by multiplying the time difference and acoustic speed. Its accuracy is vulnerable to multiple factors such as time synchronization, multipath effect and stratification effect. In SLSMP [14] and STSL [15], time synchronization is first performed to get clock skew and offset between beacon nodes and target nodes. Hence, the distance between two synchronized nodes can be estimated precisely. An alternative approach to time synchronization is using half of the round-trip time as the propagation delay [16]. This method eliminates the impact of clock offset, but increases the communication overhead. Beniwal et al. [17] proposed a time synchronization-free algorithm. They assumed that beacon nodes dive and rise in a vertical direction. Sensor nodes passively receive two localization messages from one beacon node and estimate the distance to the beacon node geometrically. In [18], an EM algorithm is proposed to alleviate the degradation caused by multipath effect, in which line-of-sight (LOS) and non line-of-sight (NLOS) links are identified to support subsequent localization. Considering the acoustic speed varies with depths, a ranging method with stratification effect compensation is put forward in [19], in which bias-free distance estimation can be obtained through integrating the function of depth. These methods are aimed at improving the ranging accuracy.

Once the distances to reference nodes are determined, a localization algorithm is executed to convert the distance estimations into the node location. In RLS [20], a new message exchange mechanism is designed to enable fast response to events and reduce communication overhead. The nodes that detect an event send messages to a sink station, wherein the node locations are estimated by trilateration. 3DUL [21] projects three neighbor beacon nodes of each unknown node onto a horizontal plane, and conduct trilateration when four triangles in the plane are all robust. This method has high energy consumption due to two-way TOA. In [22], a novel hybrid network DR-OSN is proposed, which consists of the double-head nodes deployed on the sea surface and the moored underwater nodes linked to double-head nodes with mooring lines. The moored underwater nodes are first localized by leveraging the free drifting movement of their surface nodes with GPS module and then turn into beacon nodes to localize other underwater nodes and the floating nodes without GPS module. The whole localization process is based on trilateration and does not need presence of designated beacon nodes. Besides trilateration, Bian et al. [23] proposed a hyperbola-based approach to eliminate the localization ambiguity existing in trilateration. MP-PSO [24] searches the locations of the beacon nodes using particle swarm optimization technique and calculates the velocities of the unknown nodes based on the velocities of their neighboring beacons. Then, the current location of an unknown node can be estimated by adding its previous location with its velocity. In [25], MDS-MAP (C, E) algorithm is proposed, in which the distance estimations within one-hop and two-hop nodes are obtained to form a distance matrix, and then the multi-dimensional scaling is performed to transform the matrix to the nodes’ locations.

Many localization algorithms reveal the weakness when they are applied in localization for large-scale UASNs. The situation of sparse beacon nodes brings new challenges. The unknown nodes can’t be localized due to a lack of sufficient beacon nodes. SLMP [26] provides an iterative localization technique to solve this problem. The unknown nodes estimate the locations of the beacon nodes based on their mobility speed vectors and the locations received last time. The location of an unknown node is calculated by multilateration when the number of its reference nodes is more than 4. The localization confidence is then obtained based on the confidence of its reference nodes and its own localization accuracy. The localized node whose confidence value is larger than confidence threshold becomes a new reference node and helps other unknown nodes to localize themselves. MANCL [27], TPS [28] and TP-TSFL [29] adopt a similar localization strategy. In TPS, three-dimensional Euclidean distance estimation is used to compute the distances to the reference nodes within two-hop range. This method is supplemented in MANCL using communication and vote mechanisms. Furthermore, MANCL utilizes DV-HOP distance estimation to search for new reference nodes. DRL [30] presents a double rate scheme, in which low rate mode is used for estimating the distances to multi-hop reference nodes and a high rate mode helps to transmit a mass of data in the localization procedure. These algorithms improve localization coverage and have a low communication cost, but with low localization accuracy.

The algorithms mentioned above make the best of spatial correlation between nodes; however, none of them exploits temporal dependency. The characteristic of continuous movement with time can help to improve localization performance. In [31], an offline localization algorithm that takes full advantage of all available distance estimations between nodes at different time instants is proposed. The factor graph is employed to express the temporal dependency of consecutive time instants and the distance constraints on node pairs. Although this method gains high localization accuracy, it does not offer real-time localization and is not suitable for long-term UASNs. JSL [19] utilizes an IMM filter to alleviate the impact of the node mobility. It predicts the locations of the nodes based on that of previous time instant and conducts the correction according to the location measurements. Two filters running in parallel are used, which are Kalman filter for uniform moving and extended Kalman filter (EKF) for maneuvering, respectively. The final location estimation comes from the combination of the estimations from the two filters. The drawback of JSL is that the location measurements may introduce additional errors and EKF has a possibility of divergence. SLSMP [14] alleviates localization error by applying Kalman filter and averaging filter. It performs localization only when the target node locates at fixed sending point, which has no universality.

## 3. Network Model and Problem Definition

In this section, we first describe the network model and then give the definition of the localization problem in the model.

### 3.1. Network Model

**Figure 2:**
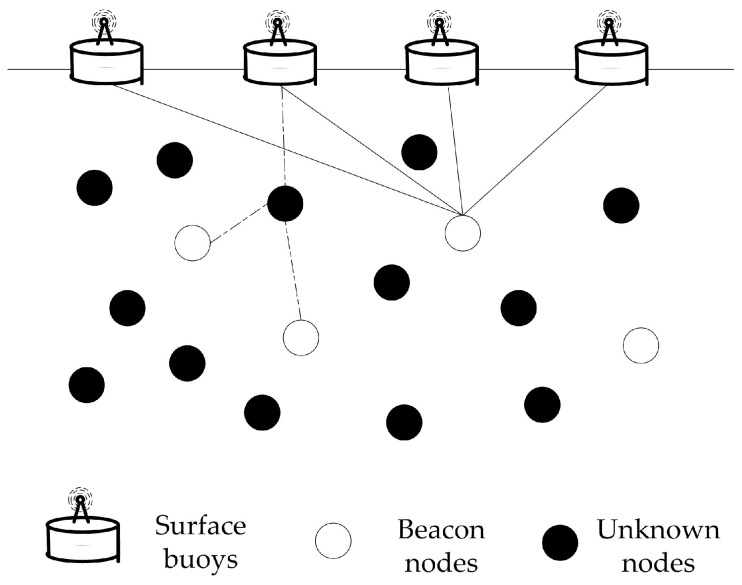
Network model.

Figure 2 shows the network architecture that is commonly adopted in previous works [24,26,32,33]. It is assumed that there are three types of nodes: surface buoys, beacon nodes and unknown nodes. Surface buoys float on the water surface and are able to obtain their locations by use of the equipped GPS. Beacon and unknown nodes are randomly distributed in the deployment area. Beacon nodes have the ability to communicate with surface buoys directly and perform self-localization since they are more powerful compared to unknown nodes. In practice, beacon nodes may be Autonomous Underwater Vehicles (AUVs) or super sensor nodes. Unknown nodes have limited energy and computation ability. They can only communicate directly with neighbor nodes and passively listen to beacon signals, thus the energy consumption can be reduced. With the help of the beacon nodes, their locations can be estimated in a distributed manner. Furthermore, the localization process is run iteratively to alleviate the impact of insufficient beacon nodes. Some unknown nodes that have been localized are selected as the reference nodes to help with other unknown nodes’ localization.

Traditional 3D localization algorithms for UASNs require at least four non-coplanar reference nodes within the communication range of one unknown node. This requires a high node density in the deployment area and thus results in an increase of the overall cost. To solve this problem, in this paper, the 3D localization problem is simplified to a 2D counterpart using a projection technique [21,34]. With the depth information acquired from cheap pressure sensors, one unknown node can project its neighbor reference nodes onto the horizontal plane of its depth. The localization can then be conducted on a 2D plane, with the requirement of at least three non-collinear reference nodes.

### 3.2. Problem Statement

Consider an UASN with N number of nodes, in which there are B beacon nodes with the identifiers {1,2,…,B} and *U* unknown nodes with the identifiers {B+1,B+2,…,B+U}. The localization procedures for all unknown nodes are similar in the distributed context. For simplicity, we consider a specific unknown node *i* with the location of $s_{t}$ at time instant *t*, in which the superscript *i* is suppressed, as well as in what follows. The transition of its location is characterized by first-order Markov model $s_{t}∼p\left(s_{t}|s_{t-1}\right)$, i.e., $s_{t}$ is only related with $s_{t-1}$ and independent of $\left\{s_{0},s_{1},\ldots ,s_{t-2}\right\}$. The coarse estimation of $s_{t}$ can be obtained based on $s_{t-1}$, which is regarded as a priori information of subsequent procedure. Then, the location estimation is refined by incorporating spatial correlation with its neighbor reference nodes. The reference node set consists of the neighbor nodes that have been localized with high confidence, which are denoted by $\left\{s^{j}|\;||s_{t}^{j}-s_{t}||\leq R_{j},\eta_{t}^{j}\geq \delta ,\forall j=1,\ldots ,i-1,i+1,\ldots ,N\right\}$, where $R_{j}$ indicates the communication range of the node *j*, $\eta_{t}^{j}$ denotes the confidence value of the location estimation of node *j* and $\bar{\delta }$ is the predefined confidence threshold. These reference nodes broadcast beacon signals. Once the signal arrives at the unknown node, the measurement $z_{t}^{k}$ between the unknown node and beacon node *k* can be obtained. Considering that the redundant measurements may incur additional errors, we sort the measurements according to the updated confidence value (see Section 4.5) and select the former $N_{r}$ nodes to form a final reference node set $R_{t}$. All the measurements from the reference nodes in $R_{t}$ form the spatial observation $z_{t}=\left\{z_{t}^{k},\forall s^{k}\in R_{t}\right\}$. Given the temporal and spatial observations, the localization procedure can be treated as a Bayesian filtering problem, which consists of prediction and update phases as follows:

Prediction:

(1) $$p\left(s_{t}|z_{1:t-1}\right)=\int p\left(s_{t}|s_{t-1}\right)p\left(s_{t-1}|z_{1:t-1}\right)\;ds_{t-1},$$

Update:

(2) $$p\left(s_{t}|z_{1:t}\right)=\frac{p\left(z_{t}|s_{t}\right)p\left(s_{t}|z_{1:t-1}\right)}{p(z_{t}|z_{1:t-1})},$$

where

$$p\left(z_{t}|z_{1:t-1}\right)=\int p\left(z_{t}|s_{t}\right)p\left(s_{t}|z_{1:t-1}\right)\;ds_{t}.$$

Once the location estimation of the unknown node is obtained, it is labeled as a reference node if its confidence value is higher than the confidence threshold $\bar{\delta }$. Then, it can provide spatial correlation information for its neighbor unknown nodes. Hence, the localization process of the entire network can be conducted iteratively and the locations of the unknown nodes at time instant *t* $S_{t}=\left\{s_{t}^{j}|\forall \;j=B+1,B+2,\ldots ,B+U\right\}$ can be determined.

## 4. Spatial–Temporal Variational Filtering Design

As described in Section 3, in the Bayesian framework, the estimation of $S_{t}$ requires a recursive update of posterior distribution of each unknown node. The estimation accuracy depends on the appropriate definition of the temporal evolution model of the state transition $p(s_{t}|s_{t-1})$ and the spatial measurement model $p(z_{t}|s_{t})$. In particular, the definition of the two models needs take the irregularity of the continuous mobility and the spatial-temporal variation of the ambient noises into account.

### 4.1. State Evolution Model

Considering an accurate mobility model may cause the degradation of localization performance due to the deviation of the actual motion of nodes, in this paper, we employ a General State Evolution Model [35,36,37] to fully characterize the complex mobility dynamics of the nodes. The state of the unknown node is represented by its location and velocity $s_{t}={\left[\begin{array}{c} x\left(t\right)\;v_{x}\left(t\right)\;y\left(t\right)\;v_{y}\left(t\right) \end{array}\right]}^{T}$, where (x(t),y(t)) denote the locations of the unknown node in the *x*- and *y*-axis directions, and $\left(v_{x}\left(t\right),v_{y}\left(t\right)\right)$ denote the velocities of the unknown node in the *x*- and *y*-axis directions. The necessity of incorporating the velocity in the state of a node comes from the calculation of the confidence value of its measurements, as we will see later. It is important to note that there are no constraints on the velocities and directions of the unknown node, i.e., all the information that is available in the localization process are from the spatial and temporal observations.

In the state evolution model, the current state $s_{t}$ is assumed to be Gaussian distributed with the expectation vector $\mu_{t}$ and precision matrix $\lambda_{t}$, that is, $s_{t}∼N\left(s_{t}|\mu_{t},\lambda_{t}\right)$. The expectation and precision are both assumed to be random variables, for capturing the uncertainty of the state and reflecting the dynamics of underwater environments as much as possible. The expectation $\mu_{t}$ follows a Gaussian distribution $\mu_{t}∼N\left(\mu_{t}|\mu_{t-1},\bar{\Lambda }\right)$, which means that $\mu_{t}$ transits from the expectation $\mu_{t-1}$ of the previous state in a completely random manner and the transition uncertainty is controlled by the precision matrix $\bar{\Lambda }$. For the convenience of recursively computing the posterior distribution in subsequent variational inference, the precision matrix $\lambda_{t}$ of the state is assumed to follow a conjugate Wishart distribution with the degrees of freedom $\bar{n}$ and the precision $\bar{V}$, in which the uncertainty of the state $s_{t}$ around its expectation $\mu_{t}$ is captured. In summary, the state evolution model can be represented as follows:

(3) $$\left\{\begin{array}{c} s_{t}\;∼N\left(\mu_{t},\lambda_{t}\right),\\ \mu_{t}\;∼N\left(\mu_{t-1},\bar{\Lambda }\right),\\ \lambda_{t}\;∼W_{d}\left(\bar{W},\bar{n}\right), \end{array}\right.$$

where the state $s_{t}$ is augmented with its expectation $\mu_{t}$ and precision $\lambda_{t}$, and the fixed parameters $\bar{\Lambda }$, $\bar{W}$ and $\bar{n}$ collaboratively determine the ability to model the state transition between two successive time instants. Based on this model, the probability distribution of the state $s_{t}$ can be obtained according to the Equation (1), and serve as the a priori distribution of the following procedure.

### 4.2. Measurement Model

In the measurement model, the coarse estimation of the state is refined with the spatial observations, which mainly consist of the measurements from the neighbor reference nodes in $R_{t}$. Generally, a one-way TOA method is adopted to produce the measurements due to two considerations: (1) it needs no additional hardware and thus saves costs greatly; (2) the unknown nodes passively listen to the beacon signals and only need a simple calculation, resulting in remarkable energy consumption reduction.

Supposing that the time synchronization has been achieved in the whole network, the traditional TOA method estimates the distances from the unknown node to its neighbor reference nodes by $(T_{R}-T_{S})\;\times \;v$, where $T_{S}$ and $T_{R}$ denote the sending and receiving time of the acoustic signal, respectively, and *v* is a constant speed value predetermined. However, considering the varying speed and non-straight propagation, the traditional TOA method often suffers from large errors and causes the degradation of the localization performance. Alternatively, in STVF, we use the time difference as the measurement:

(4) $$z_{t}^{j}=\Delta_{t}^{j}\;+\;\epsilon_{t}^{j},$$

where $z_{t}^{j}$ denotes the value of time difference from the unknown node to the j-th reference node, and $\epsilon_{t}^{j}$ denotes the measurement noise that follows a Gaussian distribution with zero mean and precision $\zeta_{t}$, namely, $\epsilon_{t}^{j}∼N\left(\epsilon_{t}^{j}|0,\zeta_{t}\right)$. Hence, the measurement follows $z_{t}^{j}∼N\left(z_{t}^{j}|\Delta_{t}^{j},\zeta_{t}\right)$. The actual propagation delay $\Delta_{t}^{j}$ depends on the distance between the unknown node and the j-th reference node $r_{t}^{j}$ and current acoustic speed $v_{t}$, i.e., $\Delta_{t}^{j}=\frac{r_{t}^{j}}{v_{t}}$. The distance $r_{t}^{j}$ can be further represented with $r_{t}^{j}\left(s_{t}\right)=||A\left(s_{t}-s_{t}^{j}\right){\left|\right|}_{2}$, where $s_{t}^{j}$ stands for the state of the j-th reference node and A is a matrix for selecting the location information from $s_{t}$ and $s_{t}^{j}$:

$$A=\left[\begin{array}{cccc} 1 & 0 & 0 & 0\\ 0 & 0 & 0 & 0\\ 0 & 0 & 1 & 0\\ 0 & 0 & 0 & 0 \end{array}\right].$$

The speed $v_{t}$ is assumed to be Gaussian distributed with the expectation $\bar{v}$ and the precision $\bar{V}$, namely, $v_{t}∼N\left(v_{t}|\bar{v},\bar{V}\right)$. This a priori information reflects the time-varying characteristic of the propagation speed and implies that the propagation speed within the communication range of the unknown node is assumed to be same. It also helps to compensate the measurement error caused by other uncertain factors such as multipath effect and stratification effect. To further capture the uncertainty of the measurements, the precision $\zeta_{t}$ of the Gaussian variable $z_{t}^{j}$ is naturally assumed to be the conjugate Gamma distribution $\zeta_{t}∼Gam\left(\zeta_{t}|\bar{\alpha },\bar{\beta }\right)$, where $\bar{\alpha }$ and $\bar{\beta }$ are the shape and scale parameters, respectively. In summary, the uncertainty of the measurements can be captured by the randomness of the acoustic speed and the precision scalar:

(5) $$\left\{\begin{array}{c} z_{t}^{j}\;∼N\left(\frac{r_{t}^{j}\left(s_{t}\right)}{v_{t}},\zeta_{t}\right),\\ v_{t}\;∼N\left(\bar{v},\bar{V}\right),\\ \zeta_{t}\;∼Gam\left(\bar{\alpha },\bar{\beta }\right), \end{array}\right.$$

where the fixed parameters $\bar{v}$, $\bar{V}$, $\bar{\alpha }$ and $\bar{\beta }$ provide the ability to model the complex dynamics of underwater environments.

### 4.3. Variational Inference

Given the state evolution model on Equation (3) and the measurement model on Equation (5), the original state $s_{t}$ is extended to an augmented state $\phi_{t}=\left\{s_{t},\mu_{t},\lambda_{t},v_{t},\zeta_{t}\right\}$. Accordingly, the prediction and posterior distributions have the form of $p(\phi_{t}|Z_{1:t-1})$ and $p(\phi_{t}|Z_{1:t})$, respectively. However, the analytic solutions of the posterior distribution are intractable due to the nonlinear function of the state in the measurement model. Alternatively, we seek to find a decomposable distribution $q(\phi_{t})$ to approximate the posterior distribution $p(\phi_{t}|Z_{1:t})$ using a variational approach, in which the Kullback–Leibler (KL) divergence between the two distributions is minimized:

(6) $$KL\left(q||p\right)=\int q\left(\phi_{t}\right)ln\left\{\frac{q(\phi_{t})}{p(\phi_{t}|z_{1:t})}\right\}\;d\phi_{t},$$

where

$$q\left(\phi_{t}\right)=\prod_{i}q\left(\phi_{t}^{i}\right)=q\left(x_{t}\right)q\left(\mu_{t}\right)q\left(\lambda_{t}\right)q\left(v_{t}\right)q\left(\zeta_{t}\right).$$

Under the condition of maximizing the log-likelihood of the measurement data $p(Z_{1:t})$, the minimization of KL divergence is equivalent to the maximization of the lower bound of $p(Z_{1:t})$ [38]:

(7) $$L\left(q\right)=\int q\left(\phi_{t}\right)ln\left\{\frac{p(\phi_{t},z_{1:t})}{q(\phi_{t})}\right\}\;d\phi_{t}.$$

We then substitute the factorized form of $q(\phi_{t})$ into Equation (7) and conduct optimization with respect to each distribution in turns:

$$\begin{array}{cc} L(q) & =\int \prod_{i}q\left(\phi_{t}^{i}\right)\left\{ln\;p\left(\phi_{t},z_{1:t}\right)-\sum_{i}ln\;q\left(\phi_{t}^{i}\right)\right\}\;d\phi_{t}\\ & =\int q\left(\phi_{t}^{j}\right)\left\{\int ln\;p\left(\phi_{t},z_{1:t}\right)\prod_{i\neq j}q\left(\phi_{t}^{i}\right)\;d\phi_{t}^{i}\right\}d\phi_{t}^{j}-\int q\left(\phi_{t}^{j}\right)ln\;q\left(\phi_{t}^{j}\right)d\phi_{t}^{j}+Const\\ & =\int q\left(\phi_{t}^{j}\right){\langle ln\;p\left(\phi_{t},z_{1:t}\right)\rangle }_{\prod_{i\neq j}}\;d\phi_{t}^{j}-\int q\left(\phi_{t}^{j}\right)ln\;q\left(\phi_{t}^{j}\right)d\phi_{t}^{j}+Const, \end{array}$$

where ${\langle •\rangle }_{q(\theta )}$ denotes an expectation with respect to the q(θ) distribution. Suppose we keep the distributions $q{\left(\phi_{t}^{i}\right)}_{i\neq j}$ fixed, L(q) can be regarded as a negative KL divergence between $q(\phi_{t}^{j})$ and $exp{\langle ln\;p\left(\phi_{t},z_{1:t}\right)\rangle }_{\prod_{i\neq j}q\left(\phi_{t}^{i}\right)}$. The minimization of this KL divergence produces the optimal solution of $q(\phi_{t}^{j})$:

(8) $$q^{*}\left(\phi_{t}^{j}\right)\propto exp{\langle ln\;p\left(\phi_{t},z_{1:t}\right)\rangle }_{\prod_{i\neq j}q\left(\phi_{t}^{i}\right)}$$

This means the optimal solution is obtained by considering the log of the joint distribution over both the state and observation variables and then taking the expectation with respect to $q{\left(\phi_{t}^{i}\right)}_{i\neq j}$.

### 4.4. Variational Prediction

In the prediction phase, we seek to find a coarse estimation of the current state $\phi_{t}$ based on previous state $\phi_{t-1}$ and the state evolution model on Equation (3). Assume we have obtained the approximate distribution $q(\phi_{t-1})$ at time instant t−1, according to Equation (1), the predictive distribution of the current state can be given as:

(9) $$p\left(\phi_{t}|z_{1:t-1}\right)\propto \int p\left(\phi_{t|t-1}|\phi_{t-1}\right)q\left(\phi_{t-1}\right)\;d\phi_{t-1},$$

where $p(\phi_{t}|\phi_{t-1})$ means the transition distribution from the previous state to the current state. We substitute the definition of each state variable into the transition distribution and rewrite it as:

$$\begin{array}{cc} p(\phi_{t}|\phi_{t-1}) & =p(s_{t},\mu_{t},\lambda_{t},v_{t},\zeta_{t}|s_{t-1},\mu_{t-1},\lambda_{t-1},v_{t-1},\zeta_{t-1})\\ & =p(s_{t}|\mu_{t},\lambda_{t},v_{t},\zeta_{t},s_{t-1},\mu_{t-1},\lambda_{t-1},v_{t-1},\zeta_{t-1})\\ & \;\;\;\;p(\mu_{t}|\lambda_{t},v_{t},\zeta_{t},s_{t-1},\mu_{t-1},\lambda_{t-1},v_{t-1},\zeta_{t-1})\\ & \;\;\;\;p(\lambda_{t}|v_{t},\zeta_{t},s_{t-1},\mu_{t-1},\lambda_{t-1},v_{t-1},\zeta_{t-1})\\ & \;\;\;\;p(v_{t}|\zeta_{t},s_{t-1},\mu_{t-1},\lambda_{t-1},v_{t-1},\zeta_{t-1})\\ & \;\;\;\;p(\zeta_{t}|s_{t-1},\mu_{t-1},\lambda_{t-1},v_{t-1},\zeta_{t-1})\\ & =p\left(s_{t}|\mu_{t},\lambda_{t}\right)p\left(\mu_{t}|\mu_{t-1}\right)p\left(\lambda_{t}\right)p\left(v_{t}\right)p\left(\zeta_{t}\right). \end{array}$$

Considering the constraint $\int q\left(\phi_{t-1}\right)\;d\phi_{t-1}=\prod_{i}\int q\left(\phi_{t-1}^{i}\right)d\phi_{t-1}^{i}=1$, the predictive distribution takes the form as follows:

(10) $$\begin{array}{cc} p(\phi_{t}|z_{1:t-1}) & \propto \int p\left(s_{t}|\mu_{t},\lambda_{t}\right)p\left(\mu_{t}|\mu_{t-1}\right)p\left(\lambda_{t}\right)p\left(v_{t}\right)p\left(\zeta_{t}\right)q\left(x_{t-1}\right)q\left(\mu_{t-1}\right)q\left(\lambda_{t-1}\right)q\left(\zeta_{t-1}\right)\;d\phi_{t-1}\\ & \propto p\left(s_{t}|\mu_{t},\lambda_{t}\right)p\left(\lambda_{t}\right)p\left(v_{t}\right)p\left(\zeta_{t}\right)q_{p}\left(\mu_{t}\right), \end{array}$$

where $q_{p}\left(\mu_{t}\right)=\int p\left(\mu_{t}|\mu_{t-1}\right)q\left(\mu_{t-1}\right)\;d\mu_{t-1}$. Hence, the current state only depends on the variable $\mu_{t-1}$ of the previous state through the transition distribution $p(\mu_{t}|\mu_{t-1})$. Assume $q(\mu_{t-1})$ is Gaussian distributed, $q\left(\mu_{t-1}\right)=N\left(\mu_{t-1}|\mu_{t-1}^{*},\Lambda_{t-1}^{*}\right)$, the marginal distribution $q_{p}\left(\mu_{t}\right)$ of the joint Gaussian distribution $p\left(\mu_{t}|\mu_{t-1}\right)q\left(\mu_{t-1}\right)$ is also a Gaussian distribution:

(11) $$q_{p}\left(\mu_{t}\right)=N\left(\mu_{t}|\mu_{t}^{p},\Lambda_{t}^{p}\right),$$

where the expectation vector $\mu_{t}^{p}=\mu_{t-1}^{*}$ and the precision matrix $\Lambda_{t}^{p}={\left(\Lambda_{t-1}^{*\;-1}+{\bar{\Lambda }}^{-1}\right)}^{-1}$. Now the exact forms of all the components of $p(\phi_{t}|z_{1:t-1})$ have been determined, and we can approximate it with the distribution $q_{t|t-1}\left(\phi_{t}\right)$ using the variational approach. According to Equation (8), the distribution $q_{t|t-1}\left(\mu_{t}\right)$ is inferred as follows:

(12) $$\begin{array}{cc} q_{t|t-1}\left(\mu_{t}\right) & \propto exp{\langle ln\;p\left(\phi_{t},z_{1:t-1}\right)\rangle }_{q_{t|t-1}\left(s_{t}\right)q_{t|t-1}\left(\lambda_{t}\right)q_{t|t-1}\left(v_{t}\right)q_{t|t-1}\left(\zeta_{t}\right)}\\ & \propto exp{\langle ln\;p\left(\phi_{t}|z_{1:t-1}\right)\rangle }_{q_{t|t-1}\left(s_{t}\right)q_{t|t-1}\left(\lambda_{t}\right)q_{t|t-1}\left(v_{t}\right)q_{t|t-1}\left(\zeta_{t}\right)}\\ & \propto exp\langle ln\;p\left(x_{t}|\mu_{t},\lambda_{t}\right)+ln\;p\left(\lambda_{t}\right)+ln\;p\left(v_{t}\right)+ln\;p\left(\zeta_{t}\right)+\\ & \;\;\;\;ln\;q_{p}\left(\mu_{t}\right)\rangle_{q_{t|t-1}\left(s_{t}\right)q_{t|t-1}\left(\lambda_{t}\right)q_{t|t-1}\left(v_{t}\right)q_{t|t-1}\left(\zeta_{t}\right)}\\ & \propto q_{p}\left(\mu_{t}\right)exp{\langle ln\;p\left(s_{t}|\mu_{t},\lambda_{t}\right)\rangle }_{q_{t|t-1}\left(s_{t}\right)q_{t|t-1}\left(\lambda_{t}\right)}. \end{array}$$

It is observed that the approximate distribution $q_{t|t-1}\left(\mu_{t}\right)$ only depends on the state variables $s_{t}$ and $\lambda_{t}$. By substituting Equations (3) and (11) into Equation (12), we have:

$$\begin{array}{cc} q_{t|t-1}\left(\mu_{t}\right) & \propto N\left(\mu_{t}|\mu_{t}^{p},\Lambda_{t}^{p}\right)exp{\langle -\frac{1}{2}{\left(s_{t}-\mu_{t}\right)}^{T}\lambda_{t}\left(s_{t}-\mu_{t}\right)\rangle }_{q_{t|t-1}\left(s_{t}\right)q_{t|t-1}\left(\lambda_{t}\right)}\\ & \propto N\left(\mu_{t}|\mu_{t}^{p},\Lambda_{t}^{p}\right)exp-\frac{1}{2}\left\{tr\left[{\langle \lambda_{t}\rangle }_{q_{t|t-1}\left(\lambda_{t}\right)}{\langle {\left(s_{t}-\mu_{t}\right)}^{T}\left(s_{t}-\mu_{t}\right)\rangle }_{q_{t|t-1}\left(s_{t}\right)}\right]\right\}\\ & \propto N\left(\mu_{t}|\mu_{t}^{p},\Lambda_{t}^{p}\right)N\left(\mu_{t}|{\langle s_{t}\rangle }_{t|t-1},{\langle \lambda_{t}\rangle }_{t|t-1}\right), \end{array}$$

where the subscript $q_{t|t-1}\left(•\right)$ is suppressed as t|t−1. Hence, the predictive variable $\mu_{t}$ follows a Gaussian distribution $\mu_{t}∼N\left(\mu_{t|t-1}^{*},\Lambda_{t|t-1}^{*}\right)$. Its expectation vector and precision matrix is derived as:

(13) $$\begin{array}{c} \mu_{t|t-1}^{*}=\Lambda_{t|t-1}^{*-1}\left({\langle \lambda_{t}\rangle }_{t|t-1}{\langle s_{t}\rangle }_{t|t-1}+\Lambda_{t}^{p}\mu_{t}^{p}\right),\\ \Lambda_{t|t-1}^{*}={\langle \lambda_{t}\rangle }_{t|t-1}+\Lambda_{t}^{p}. \end{array}$$

Similar with the deduction process in Equation (12), the distribution $q_{t|t-1}\left(\lambda_{t}\right)$ can be approximated as:

$$\begin{array}{cc} q_{t|t-1}\left(\lambda_{t}\right) & \propto p\left(\lambda_{t}\right)exp{\langle ln\;p\left(s_{t}|\mu_{t},\lambda_{t}\right)\rangle }_{q_{t|t-1}\left(s_{t}\right)q_{t|t-1}\left(\mu_{t}\right)}\\ & \propto W_{d}\left(\bar{V},\bar{n}\right){\left|\lambda_{t}\right|}^{\frac{1}{2}}exp-\frac{1}{2}\left\{tr\left[\lambda_{t}{\langle {\left(s_{t}-\mu_{t}\right)}^{T}\left(s_{t}-\mu_{t}\right)\rangle }_{q_{t|t-1}\left(s_{t}\right)q_{t|t-1}\left(\mu_{t}\right)}\right]\right\}\\ & \propto |\lambda_{t}{|}^{\frac{(\bar{n}+1)-d-1}{2}}exp-\frac{1}{2}\left\{tr\left[\lambda_{t}\left({\langle s_{t}s_{t}^{T}\rangle }_{t|t-1}-{\langle s_{t}\rangle }_{t|t-1}{\langle \mu_{t}\rangle }_{t|t-1}^{T}-{\langle \mu_{t}\rangle }_{t|t-1}{\langle s_{t}\rangle }_{t|t-1}^{T}+{\langle \mu_{t}\mu_{t}^{T}\rangle }_{t|t-1}+{\bar{V}}^{-1}\right)\right]\right\}, \end{array}$$

which means that $\lambda_{t}$ follows a Wishart distribution $\lambda_{t}∼W_{d}\left(W_{t|t-1}^{*},n_{t|t-1}^{*}\right)$ with the parameters:

(14) $$\begin{array}{c} n_{t|t-1}^{*}=\bar{n}+1,\\ W_{t|t-1}^{*}={\left({\langle s_{t}s_{t}^{T}\rangle }_{t|t-1}-{\langle s_{t}\rangle }_{t|t-1}{\langle \mu_{t}\rangle }_{t|t-1}^{T}-{\langle \mu_{t}\rangle }_{t|t-1}{\langle s_{t}\rangle }_{t|t-1}^{T}+{\langle \mu_{t}\mu_{t}^{T}\rangle }_{t|t-1}+{\bar{W}}^{-1}\right)}^{-1}. \end{array}$$

For the variables $v_{t}$ and $\lambda_{t}$, they do not obtain additional information from the transition distribution since they are independent of $\mu_{t}$ and $\mu_{t-1}$. Therefore, the distributions of $q_{t|t-1}\left(v_{t}\right)$ and $q_{t|t-1}\left(\zeta_{t}\right)$ remain the same as $p(v_{t})$ and $p(\zeta_{t})$ in Equation (5), with the following parameters:

(15) $$\begin{array}{c} v_{t|t-1}^{*}=\bar{v},\;V_{t|t-1}^{*}=\bar{V},\\ \alpha_{t|t-1}^{*}=\bar{\alpha },\;\beta_{t|t-1}^{*}=\bar{\beta }. \end{array}$$

The distribution of the variable $s_{t}$ depends on $\mu_{t}$ and $\lambda_{t}$, which can be deduced as follows:

(16) $$\begin{array}{cc} q_{t|t-1}\left(s_{t}\right) & \propto exp{\langle ln\;p\left(s_{t}|\mu_{t},\lambda_{t}\right)\rangle }_{q_{t|t-1}\left(\mu_{t}\right)q_{t|t-1}\left(\lambda_{t}\right)}\\ & \propto exp-\frac{1}{2}\left\{tr\left[{\langle \lambda_{t}\rangle }_{t|t-1}{\langle {\left(s_{t}-\mu_{t}\right)}^{T}\left(s_{t}-\mu_{t}\right)\rangle }_{t|t-1}\right]\right\}\\ & \propto N({\langle \mu_{t}\rangle }_{t|t-1},{\langle \lambda_{t}\rangle }_{t|t-1}). \end{array}$$

From Equations (13)–(16), we can see that each component of $q_{t|t-1}\left(\phi_{t}\right)$ depends on the expectations computed with respect to other components, which suggests that the parameters of each component distribution can be optimized iteratively. Nevertheless, we delay the iterative optimization to the following update phase because the spatial observations can be incorporated to refine the inferred locations of the unknown node.

### 4.5. Variational Update

In the measurement model, the measurement $z_{t}^{j}$ is actually time of flight from the reference node $s^{j}$ to the unknown node, which provides the spatial information to update the predicted variables $s_{t}$, $v_{t}$ and $\zeta_{t}$. The overall spatial information provided by all the reference nodes in $R_{t}$ can be represented with the joint likelihood distribution:

(17) $$p\left(z_{t}|s_{t},v_{t},\zeta_{t}\right)=\prod_{i=1}^{N_{r}}N\left(z_{t}^{i}|\frac{r_{t}^{i}\left(s_{t}\right)}{v_{t}},\zeta_{t}\right),$$

where $N_{r}$ denotes the number of the reference nodes in $R_{t}$. Next, we treat the predictive distribution $q_{t|t-1}\left(\phi_{t}\right)$ as a priori information, fuse it with the likelihood information and derive the posterior distribution as:

$$\begin{array}{cc} p(\phi_{t}|z_{1:t}) & \propto p\left(z_{t}|\phi_{t}\right)q_{t|t-1}\left(\phi_{t}\right)\\ & \propto p\left(z_{t}|s_{t},v_{t},\zeta_{t}\right)q_{t|t-1}\left(\phi_{t}\right), \end{array}$$

where the distribution form of $q_{t|t-1}\left(\phi_{t}\right)$ has been deduced in the prediction phase. Let $q_{t|t}\left(\phi_{t}\right)$ denotes the approximate posterior distribution. It is observed that the measurement $z_{t}$ is independent of the variables $\lambda_{t}$ and $\mu_{t}$, thus the likelihood function does not contain the information for further updating them. The distributions of $q_{t|t}\left(\mu_{t}\right)$ and $q_{t|t}\left(\lambda_{t}\right)$ remain the same as $q_{t|t-1}\left(\mu_{t}\right)$ and $q_{t|t-1}\left(\lambda_{t}\right)$, with the following parameters:

(18) $$\begin{array}{c} \mu_{t|t}^{*}=\Lambda_{t|t}^{*-1}\left({\langle \lambda_{t}\rangle }_{t|t}{\langle s_{t}\rangle }_{t|t}+\Lambda_{t}^{p}\mu_{t}^{p}\right),\\ \Lambda_{t|t}^{*}={\langle \lambda_{t}\rangle }_{t|t}+\Lambda_{t}^{p},\\ n_{t|t}^{*}=\bar{n}+1,\\ W_{t|t}^{*}={\left({\langle s_{t}s_{t}^{T}\rangle }_{t|t}-{\langle s_{t}\rangle }_{t|t}{\langle \mu_{t}\rangle }_{t|t}^{T}-{\langle \mu_{t}\rangle }_{t|t}{\langle s_{t}\rangle }_{t|t}^{T}+{\langle \mu_{t}\mu_{t}^{T}\rangle }_{t|t}+{\bar{W}}^{-1}\right)}^{-1}. \end{array}$$

The involved expectations have the expressions:

(19) $$\begin{array}{c} {\langle \mu_{t}\rangle }_{t|t}=\mu_{t|t}^{*},\\ {\langle \mu_{t}\mu_{t}^{T}\rangle }_{t|t}={\langle \mu_{t}\rangle }_{t|t}{\langle \mu_{t}\rangle }_{t|t}^{T}+\Lambda_{t|t}^{*-1},\\ {\langle \lambda_{t}\rangle }_{t|t}=n_{t|t}^{*}W_{t|t}^{*}. \end{array}$$

The remaining expectations involve the joint optimization of the component distributions $q_{t|t}\left(s_{t}\right)$ and $q_{t|t}\left(v_{t}\right)$, which can be derived with the same procedure as Equation (12):

$$\begin{array}{cc} q_{t|t}\left(s_{t}\right) & \propto q_{t|t-1}\left(s_{t}\right)exp{\langle ln\;p\left(z_{t}|s_{t},v_{t},\zeta_{t}\right)\rangle }_{q_{t|t}\left(v_{t}\right)q_{t|t}\left(\zeta_{t}\right)}\\ & \propto N\left({\langle \mu_{t}\rangle }_{t|t},{\langle \lambda_{t}\rangle }_{t|t}\right)exp{\langle \sum_{i=1}^{N_{r}}\frac{1}{2}ln\zeta_{t}-\frac{1}{2}\zeta_{t}{\left[z_{t}^{i}-\frac{r_{t}^{i}\left(s_{t}\right)}{v_{t}}\right]}^{2}\rangle }_{q_{t|t}\left(v_{t}\right)q_{t|t}\left(\zeta_{t}\right)}\\ & \propto N\left({\langle \mu_{t}\rangle }_{t|t},{\langle \lambda_{t}\rangle }_{t|t}\right)\prod_{i=1}^{N_{r}}N\left(z_{t}^{i}|\frac{r_{t}^{i}\left(s_{t}\right)}{{\langle v_{t}\rangle }_{t|t}},{\langle \zeta_{t}\rangle }_{t|t}\right), \end{array}$$

$$\begin{array}{cc} q_{t|t}\left(v_{t}\right) & \propto q_{t|t-1}\left(v_{t}\right)exp{\langle ln\;p\left(z_{t}|s_{t},v_{t},\zeta_{t}\right)\rangle }_{q_{t|t}\left(s_{t}\right)q_{t|t}\left(\zeta_{t}\right)}\\ & \propto N\left(\bar{v},\bar{V}\right)exp{\langle \sum_{i=1}^{N_{r}}\frac{1}{2}ln\zeta_{t}-\frac{1}{2}\zeta_{t}{\left[z_{t}^{i}-\frac{r_{t}^{i}\left(s_{t}\right)}{v_{t}}\right]}^{2}\rangle }_{q_{t|t}\left(s_{t}\right)q_{t|t}\left(v_{t}\right)}\\ & \propto N\left(\bar{v},\bar{V}\right)\prod_{i=1}^{N_{r}}N\left(z_{t}^{i}|\frac{r_{t}^{i}\left({\langle s_{t}\rangle }_{t|t}\right)}{v_{t}},{\langle \zeta_{t}\rangle }_{t|t}\right). \end{array}$$

We can see that the logarithm likelihood function is nonlinear over the variables $v_{t}$ and $s_{t}$, which means that the analytic solutions of $q_{t|t}\left(v_{t}\right)$ and $q_{t|t}\left(s_{t}\right)$ are intractable. Hence, we resort to an importance sampling method to compute the expectations approximately, wherein the Gaussian distribution is treated as the proposal distribution and the weights are calculated according to the likelihood function. Let ${\left\{v_{t}^{\left(k\right)},vw_{t}^{\left(k\right)}\right\}}_{k=1}^{N_{v}}$ denote the samples of $q_{t|t}\left(v_{t}\right)$ and the corresponding weights, where $v_{t}^{\left(k\right)}∼N\left(\bar{v},\bar{V}\right)$ and $vw_{t}^{\left(k\right)}\propto \prod_{i=1}^{N_{r}}N\left(z_{t}^{i}|\frac{r_{t}^{i}\left({\langle s_{t}\rangle }_{t|t}\right)}{v_{t}^{\left(k\right)}},{\langle \zeta_{t}\rangle }_{t|t}\right)$. Its expectation is given by:

(20) $$\begin{array}{c} {\langle v_{t}\rangle }_{t|t}=\sum_{k=1}^{N_{v}}vw_{t}^{\left(k\right)}v_{t}^{\left(k\right)}. \end{array}$$

For $q_{t|t}\left(s_{t}\right)$, the samples are drawn by $s_{t}^{\left(k\right)}∼N\left({\langle \mu_{t}\rangle }_{t|t},{\langle \lambda_{t}\rangle }_{t|t}\right)$ and the weights are calculated by $sw_{t}^{\left(k\right)}\propto \prod_{i=1}^{N_{r}}N\left(z_{t}^{i}|\frac{r_{t}^{i}\left(s_{t}^{\left(k\right)}\right)}{{\langle v_{t}\rangle }_{t|t}},{\langle \zeta_{t}\rangle }_{t|t}\right)$. Its associated expectation and precision are approximated as:

(21) $$\begin{array}{c} {\langle s_{t}\rangle }_{t|t}=\sum_{k=1}^{N_{s}}sw_{t}^{\left(k\right)}s_{t}^{\left(k\right)},\\ {\langle s_{t}s_{t}^{T}\rangle }_{t|t}=\sum_{k=1}^{N_{s}}sw_{t}^{\left(k\right)}s_{t}^{\left(k\right)}s_{t}^{(k)T}. \end{array}$$

Finally, the component distribution $q_{t|t}\left(\zeta_{t}\right)$ can be derived as:

$$\begin{array}{cc} q_{t|t}\left(\zeta_{t}\right) & \propto q_{t|t-1}\left(\zeta_{t}\right)exp{\langle ln\;p\left(z_{t}|s_{t},v_{t},\zeta_{t}\right)\rangle }_{q_{t|t}\left(s_{t}\right)q_{t|t}\left(v_{t}\right)}\\ & \propto Gam\left(\bar{\alpha },\bar{\beta }\right)\zeta_{t}^{\frac{N_{r}}{2}}exp{\langle \sum_{i=1}^{N_{r}}-\frac{1}{2}\zeta_{t}{\left[z_{t}^{i}-\frac{r_{t}^{i}\left(s_{t}\right)}{v_{t}}\right]}^{2}\rangle }_{q_{t|t}\left(s_{t}\right)q_{t|t}\left(v_{t}\right)}\\ & \propto \zeta_{t}^{\bar{\alpha }-1}exp\left(-\bar{\beta }\zeta_{t}\right)\zeta_{t}^{\frac{N_{r}}{2}}\prod_{i=1}^{N_{r}}exp-\frac{1}{2}\zeta_{t}{\left[z_{t}^{i}-\frac{r_{t}^{i}\left({\langle s_{t}\rangle }_{t|t}\right)}{{\langle v_{t}\rangle }_{t|t}}\right]}^{2}, \end{array}$$

which implies that the variable $\zeta_{t}$ follows a Gamma distribution, and its shape and scale parameters have the expressions:

(22) $$\begin{array}{c} \alpha_{t|t}^{*}=\bar{\alpha }+\frac{N_{r}}{2},\\ \beta_{t|t}^{*}=\bar{\beta }+\frac{1}{2}\sum_{i=1}^{N_{r}}{\left[z_{t}^{i}-\frac{r_{t}^{i}\left({\langle s_{t}\rangle }_{t|t}\right)}{{\langle v_{t}\rangle }_{t|t}}\right]}^{2}. \end{array}$$

Hence, we have:

(23) $$\begin{array}{c} {\langle \zeta_{t}\rangle }_{t|t}=\alpha_{t|t}^{*}\beta_{t|t}^{*-1}. \end{array}$$

Equations (18)–(23) suggest that these parameters can take turns being updated until convergence. The pseudo-code of the whole localization process is summarized in Algorithm 1.

**Algorithm 1:** STVF localization algorithm.

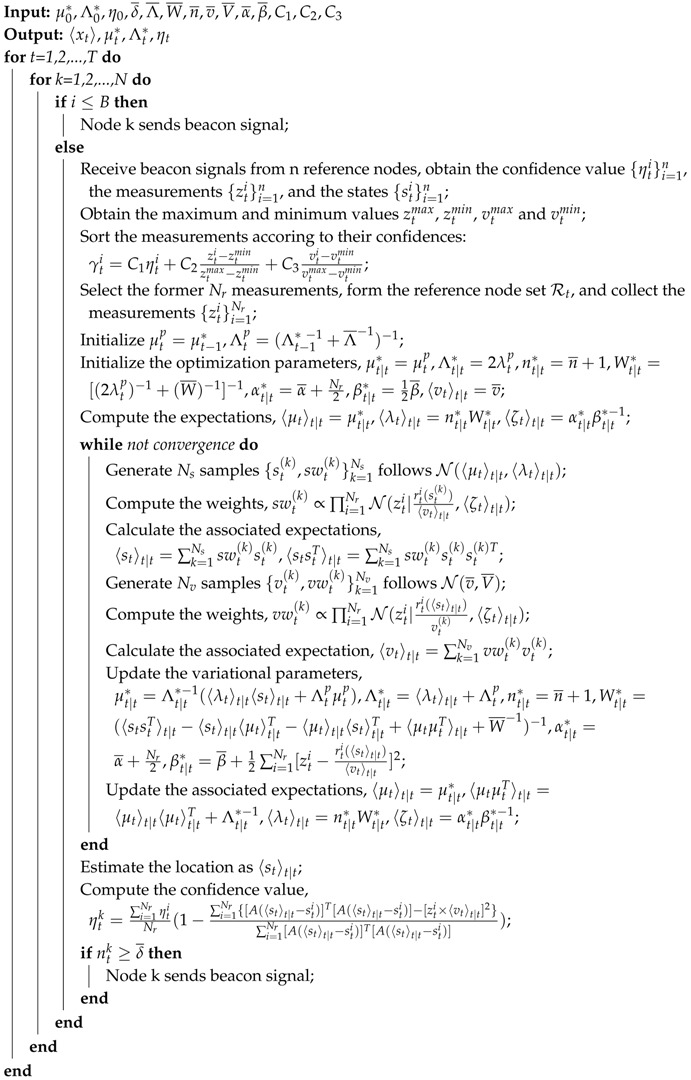



### 4.6. Iterative Localization

Considering the hardware costs and the deployment difficulties, the beacon nodes usually account for a small proportion of all the nodes in most UASNs. The network has low localization coverage, i.e., many unknown nodes can’t be localized due to lack of sufficient beacon nodes, especially for large-scale UASNs. To solve this problem, an iterative localization scheme is adopted, in which an unknown node that has been localized with high confidence can serve as a reference node and broadcast beacon signals to localize other unknown nodes. The confidence value of an unknown node is not only related to its localization error, but also related to the confidence values of its reference nodes. In STVF, the confidence value is calculated as:

(24) $$\eta =\left\{\begin{array}{cc} 1, & \;\mathrm{if}\;\mathrm{the}\;\mathrm{node}\;\mathrm{is}\;a\;\mathrm{beacon},\\ \frac{\sum_{i=1}^{N_{r}}\eta_{t}^{i}}{N_{r}}\left(1-\frac{E}{D}\right), & \;\mathrm{otherwise}, \end{array}\right.$$

where *D* represents the sum of the estimated distances to all the reference nodes:

(25) $$D=\sum_{i=1}^{N_{r}}{\left[A\left({\langle s_{t}\rangle }_{t|t}-s_{t}^{i}\right)\right]}^{T}\left[A\left({\langle s_{t}\rangle }_{t|t}-s_{t}^{i}\right)\right]$$

and *E* represents the sum of errors between the estimated and measured distances for all reference nodes:

(26) $$E=\sum_{i=1}^{N_{r}}\left\{{\left[A\left({\langle s_{t}\rangle }_{t|t}-s_{t}^{i}\right)\right]}^{T}\left[A\left({\langle s_{t}\rangle }_{t|t}-s_{t}^{i}\right)\right]-{\left[z_{t}^{i}\times {\langle v_{t}\rangle }_{t|t}\right]}^{2}\right\}.$$

If the confidence value of the localized node is higher than the confidence threshold $\bar{\delta }$, the node is labeled as a new reference node. Then, it will broadcast beacon signals to localize other unknown nodes.

The variational update procedure reveals that the measurements from the reference nodes are vital for improving localization accuracy. The confidence value of a reference node reflects its own localization accuracy, whereas the quality of its measurement to the unknown node is also subject to other factors such as distance and velocity. Herein, we consider the confidence value of each measurement based on that of a reference node. Next, we present the method for updating the confidence value of the measurements.

Intuitively, the long distance between the unknown node and the reference node will incur large errors, i.e., the measurement error increases with the distance. Similarly, the reference nodes with high velocities usually move a long distance between two consecutive time instants, which will increase the localization error as well. Thus, the confidence value of a measurement from the reference node $s_{t}^{i}$ can be defined as:

(27) $$\gamma_{t}^{i}=C_{1}\eta_{t}^{i}+C_{2}f_{z}\left(z_{t}^{i}\right)+C_{3}f_{v}\left(v_{t}^{i}\right),$$

where $C_{1}$, $C_{2}$ and $C_{3}$ are the weight values that satisfy $C_{1}+C_{2}+C_{3}=1$. $\eta_{t}^{i}$ denotes the confidence value of the *i*-th reference node. The function $f_{z}\left(z_{t}^{i}\right)$ computes the normalization value of $z_{t}^{i}$ as:

(28) $$f_{z}\left(z_{t}^{i}\right)=\frac{z_{t}^{i}-z_{t}^{min}}{z_{t}^{max}-z_{t}^{min}}$$

where $z_{t}^{min}$ and $z_{t}^{max}$ are the minimum and maximum values of all the measurements ${\left\{z_{t}^{i}\right\}}_{i=1}^{N_{r}}$. The function $f_{v}\left(v_{t}^{i}\right)$ computes the normalization value of $v_{t}^{i}$ as:

(29) $$f_{v}\left(v_{t}^{i}\right)=\frac{v_{t}^{i}-v_{t}^{min}}{v_{t}^{max}-v_{t}^{min}},$$

where $v_{t}^{min}$ and $v_{t}^{max}$ are the minimum and maximum values of the velocities of all the reference nodes. The velocity of a reference node is calculated as $\sqrt{v_{x}{\left(t\right)}^{2}+v_{y}{\left(t\right)}^{2}}$.

Based on Equations (27)–(29), the confidence values of all the measurements can be obtained. We sort the measurements according to their confidence values and select the former $N_{r}$ measurements to be used in the update phase, where $N_{r}$ denotes the threshold value of the reference nodes’ number and will be determined in the simulations.

## 5. Simulation Results

In this section, we present the simulations and evaluate the performance of the proposed STVF algorithm.

### 5.1. Simulation Settings

In our simulations, 200 sensor nodes are randomly deployed in the monitoring area with the range 500 m × 500 m. The initial proportion of the beacon nodes is set as 20%. The node density is defined as the expected number of the nodes within the communication range. All the nodes have the communication range of 80 m and the corresponding node density is 16. The acoustic signals propagate with the average speed 1500 m/s and the standard deviation 10 m/s. We assume the measurement errors increase with the distances, which follows the Gaussian distribution with the real propagation time as the expectation and the standard deviation to be two percent of the real propagation time. Each simulation lasts for 500 s. The localization period is set to 1 s. In a localization period, all the unknown nodes need to get their location estimations. Moreover, each simulation is run 50 times to relieve the impact of the deployment randomness. The simulation settings and the parameters involved in variational filtering are listed in Table 1.

In the simulations, we focus on two performance metrics: localization accuracy and localization coverage. Localization coverage is defined as the proportion of the unknown nodes that have been localized. Localization accuracy is defined as the average distance between the estimated locations and the real locations of all the localized nodes. We compare the performance of STVF with that of SLMP [26] because their simulation settings are similar. First, SLMP assumes a similar network model and employs an iterative localization scheme; second, the localization process of SLMP consists of the prediction and correction phases, which are similar to the variational prediction and update phases; third, to simulate the mobility pattern of the nodes in an ocean current field, we adopt the kinematic model proposed in SLMP, rather than a general random walk mobility model. The dimensionless velocity in the kinematical model is approximated as:

$$\left\{\begin{array}{c} V_{x}\left(t\right)=k_{1}\iota \omega sin\left(k_{2}x\right)cos\left(k_{3}y\right)+k_{1}\iota cos\left(2k_{1}t\right)+k_{4},\\ V_{y}\left(t\right)=-\iota \omega cos\left(k_{2}x\right)sin\left(k_{3}y\right)+k_{5}, \end{array}\right.$$

where the parameters $k_{1},k_{2},k_{3},\iota$ and ω are related to the underwater environments. Their value settings are listed in Table 1. Based on the above settings, STVF and SLMP are implemented using the MATLAB 2014a platform, on a computer with an Intel Core i3-6100 CPU 3.7 GHz and 8.0 GB RAM.

### 5.2. Localization Performance under Different Parameters

We evaluate the performance of STVF under different parameters, which include the threshold of the measurements’ number and the number of the samples.

#### 5.2.1. Impacts of the Threshold of the Measurements’ Number

**Figure 3:**
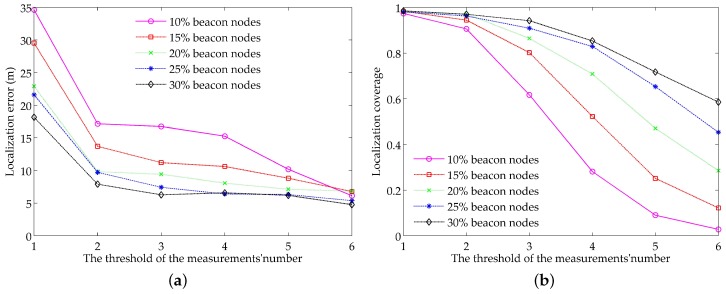
Impacts of the threshold of the measurements’ number on: (**a**) localization accuracy; (**b**) localization coverage.

As described in the problem statement, the number of the measurements has an important impact on the localization accuracy and coverage. It is necessary to determine the threshold of the measurements’ number through simulations. By keeping the parameter settings in Table 1 and setting the proportion of beacon nodes as 10%, 15%, 20%, 25% and 30%, respectively, we can get the localization results under different thresholds of the measurements’ number. The results are shown in Figure 3.

Figure 3a shows the relationship between the localization error and different thresholds. Without surprise, the localization with a high proportion of beacon nodes has a smaller error than that with a low proportion of beacon nodes. We can also see that, with the increase of the threshold number, the localization error decreases gradually. Especially, when the threshold number changes from 1 to 2, the localization error has a great decrease. In comparison, the localization error decreases a little as the threshold number changes from 2 to 3 and stabilizes eventually at some small values, except the cases of 10% and 15% beacon nodes. This can be explained as follows: most unknown nodes can be localized with a relatively high accuracy by fusing two selected measurements and their previous locations, and the localization error has no obvious decrease even if more measurements are incorporated. For the cases of 10% and 15% beacon nodes, the localization error has an obvious decrease. This is mainly because the low proportion of beacon nodes leads to that there are no redundant measurements with high confidence to be selected.

Figure 3b shows the relationship between the localization coverage and different thresholds. As expected, the localization coverage with high proportion of beacon nodes is larger than that with low proportion of beacon nodes. Moreover, the localization coverage for the cases of low proportion decreases faster because there are a few unknown nodes with sufficient reference nodes. Hence, the choice of the threshold of the measurements’ number depends on the proportion of beacon nodes and needs to consider both the localization accuracy and the localization coverage. For example, for 10% proportion of beacon nodes, the threshold can be set as 2 to ensure high localization coverage and proper localization accuracy, whereas the threshold can be set to 3 for the 30% proportion of beacon nodes, since the localization error has stabilized at small values and the localization coverage is still high. In the subsequent simulations, we select the threshold of the measurements’ number according to Figure 3.

#### 5.2.2. Impacts of the Number of the Samples

**Figure 4:**
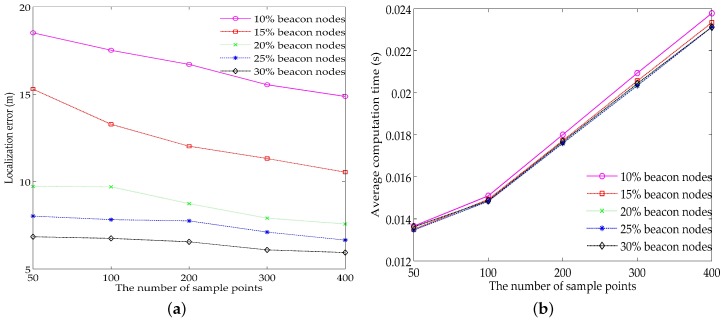
Impacts of the number of the samples on: (**a**) localization accuracy; (**b**) average computation time.

The number of the samples is another factor that can impact the localization results. Here, we simply select the same value for the samples’ number of calculating the expectations ${\langle v_{t}\rangle }_{t|t}$ and ${\langle v_{t}\rangle }_{t|t}$. By keeping the parameter settings in Table 1, choosing the threshold of the measurements’ number according to Figure 3 and changing the number of the samples from 50 to 400, the localization accuracy and the average computation time under different numbers of the sample points are shown in Figure 4.

From Figure 4a, we can observe that the localization error decreases monotonically with the increase of the samples’ number, and the degree of the error reduction also decreases with the increase of the proportion of beacon nodes. This can be explained as follows: for the low proportions of beacon nodes (10% and 15%), one unknown node usually has a limited number of neighbor reference nodes. The measurements corresponding to these reference nodes may have large deviations with real propagation delays. This means that the components of the likelihood function (17) can hardly get high probabilities simultaneously, resulting in most samples having extremely low weights. This situation can be relieved effectively by the increase of the samples’ number. In comparison, for the high proportions of beacon nodes (20%, 25% and 30%), the measurements with high confidence are selected for the localization. Thus, the samples can be generated with proper weights. The increase of its number has little contribution to the improvement of the localization accuracy. Furthermore, as shown in Figure 4b, the average computation time increases linearly with the number of the samples. Hence, simply increasing the samples’ number is of little benefit to the localization accuracy. Instead, the additional energy consumption is disadvantageous for prolonging the network lifetime.

#### 5.2.3. Localization Results

Keeping the parameter settings in Table 1, we select the threshold of the measurements’ number as 3, choose the number of the samples as 200 and then evaluate the localization results. Herein, we select the root mean square error (RMSE) as an indicator of the localization accuracy of each unknown node:

$$RMSE=\sqrt{\frac{1}{m}\sum_{t}{\left(P_{t}-P_{t}^{\prime }\right)}^{2}},$$

where *t* denotes time instants when the unknown node is localized, *m* is the number of all the time instants, $P_{t}$ is the real location of the unknown node at time instant *t* and $P_{t}^{\prime }$ is the estimated location of the unknown node at time instant *t*.

**Figure 5:**
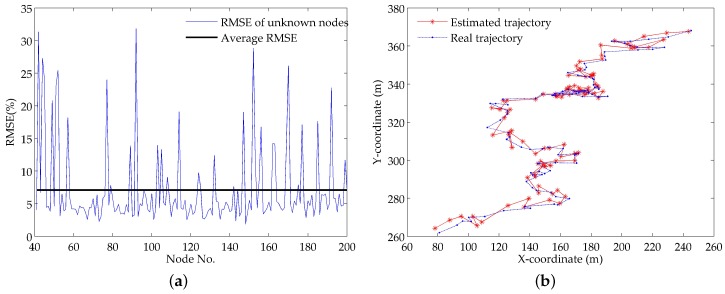
Localization results: (**a**) root mean square errors (RMSEs) of different unknown nodes; (**b**) comparison of estimated and real trajectories.

Figure 5a shows the RMSEs of all the unknown nodes in the simulation time. We can see that the RMSE of some unknown nodes has large values, which means the localization accuracy of these nodes is not very high. This is mainly because these nodes are not localized continuously. In some simulation periods, their locations can’t be estimated due to lack of sufficient reference nodes, which leads to a priori location information not being available in the next simulation period. On the whole, the localization accuracy is tolerable for this large-scale UASN. The average RMSE of all the unknown nodes is 7.09 and the RMSEs of 78.5% unknown nodes are less than the average RMSE, which means that most unknown nodes have high localization accuracy.

To illustrate the localization performance in the continuous movements with water currents, we select an unknown node with the RMSE value 5.03 and plot its real trajectory and estimated trajectory within 100 s. The unknown node starts from the coordinates (318.9, 453.8) and stops at the coordinates (383.7, 543.8). As shown in Figure 5b, the STVF algorithm can localize the node with extremely small errors in most simulation periods, even in the cases of high velocity and sharp turns. This is owing to the collaboration of the spatial correlation and temporal dependency.

**Figure 6:**
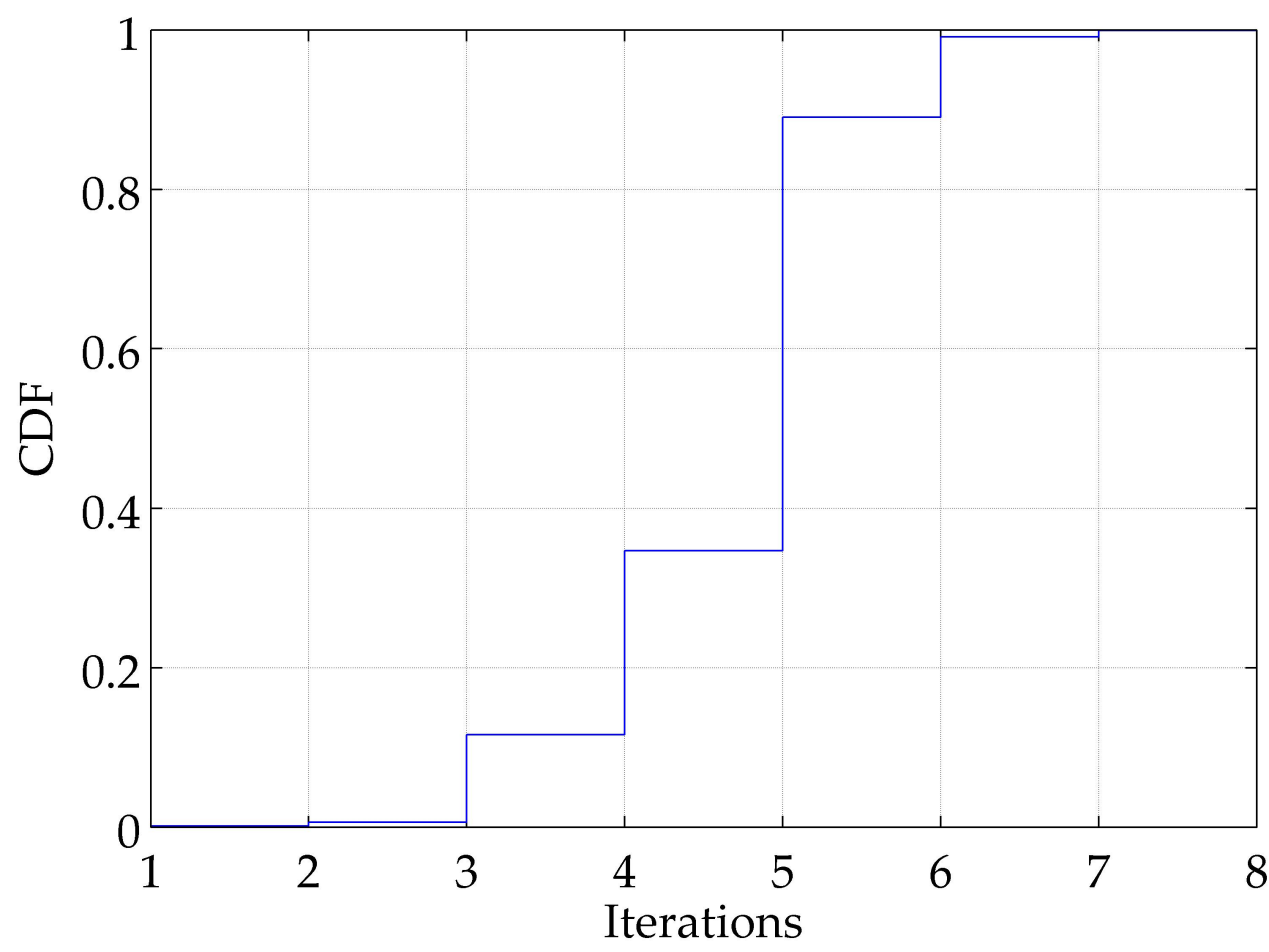
CDF of iterations.

In addition, we evaluate the proposed method on computational complexity. Although the parameters of the posterior distribution need to be optimized by turns, the optimizations mostly converge in several iteration steps. As illustrated in Figure 6, the proportion of the optimizations that converge in five iterations is up to 89.07%. All the optimizations can be accomplished in seven iterations at most and 4.64 iterations on average. Moreover, only the values of the parameters $\mu_{t-1}^{*}$ and $\Lambda_{t-1}^{*}$ of the last simulation period and the temporal optimization parameters of current simulation period need to be stored in each simulation, which indicate that limited storage memory is required. In summary, the computational complexity is acceptable and satisfiable, even in resource-constrained underwater nodes.

### 5.3. Performance Comparison with SLMP

We compare the performance of STVF with that of SLMP under different parameters, including the node density, the confidence threshold and the standard deviation of the measurements. The unique parameters of SLMP are set according to [26]. The first 100 s of each simulation is used for beacon nodes’ training. The prediction window is set as 60 s and the overlap of neighboring windows is set to be twenty percent of the prediction window. The length of the prediction step is set as 15, and the prediction error threshold is set to be five percent of the communication range.

#### 5.3.1. Impacts of the Node Density

**Figure 7:**
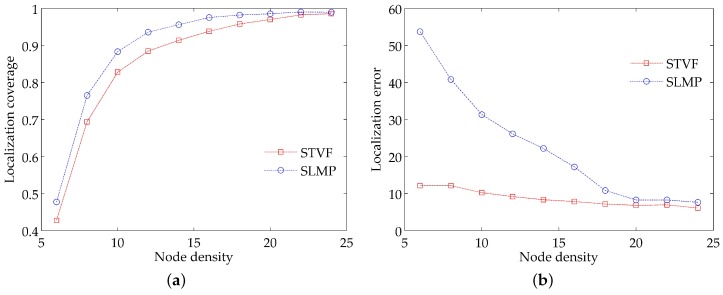
Impacts of the node density on: (**a**) localization coverage; (**b**) localization accuracy.

Keeping the parameter settings in Table 1, we set the threshold of the measurements’ number to be 2 and change the node density from 6 to 24 by adjusting the communication range of the nodes. The results for localization coverage and localization accuracy are plotted in Figure 7.

Figure 7a shows the relationship between the localization coverage and the node density. We can see that the localization coverage of STVF and SLMP both increase monotonically with the node density. Furthermore, the localization coverage of STVF is a little bit higher than that of SLMP. This is because the localizations of the unknown nodes need at least two reference nodes in STVF. For SLMP, this localizability is relaxed by two strategies: on one hand, the validity period of a reference node lasts for 60 s since its signal arrives at the corresponding unknown node, which increases greatly the number of the available reference nodes; on the other hand, an unknown node can self-estimate the current location based on the previous location and the predicted speed vector when there is more than one reference node available.

Figure 7b shows that the localization errors of STVF and SLMP both decrease with the increase of the node density. However, the decrease degree of STVF is far less than that of SLMP. When the node density is low, the network is sparse in the deployment area and the neighbor reference nodes of an unknown node are severely insufficient. In SLMP, most of the unknown nodes are localized by adding their previous locations with the predicted speed vectors, which incur large localization errors. Although STVF relaxes the requirement of the reference nodes’ number, it still maintains relatively high localization accuracy with the help of the a priori locations. As the node density increases, the reference nodes around each unknown node also increase gradually. Many unknown nodes can be localized by trilateration in SLMP, which reduces the localization error dramatically. In comparison, STVF does not change the localization strategy and thus its localization error has no obvious changes. When the node density reaches a certain point, most localizable unknown nodes get localized. The increase of the node density has little contribution to the reduction of the localization error. The localization errors of SLMP and STVF will stabilize at some small values.

#### 5.3.2. Impacts of the Confidence Threshold

**Figure 8:**
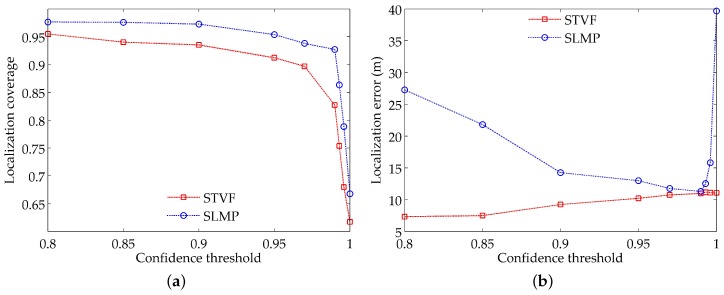
Impacts of the confidence threshold on: (**a**) localization coverage; (**b**) localization accuracy.

In this set of simulations, we keep the parameter settings in Table 1, set the threshold of the measurements’ number to be 2 and change the confidence threshold from 0.8 to 1. The results for localization coverage and localization accuracy are illustrated in Figure 8.

As shown in Figure 8a, the localization coverage of SLMP and STVF both decrease with the increase of the confidence threshold. Moreover, there exist obvious turning points for the two curves. When the confidence threshold is below the turning point value, the localization coverage decreases slowly; otherwise, the localization coverage has a sharp drop. From Figure 8a, we can observe that the turning points for SLMP and STVF are 0.97 and 0.99, respectively.

Figure 8b shows the relationship between the localization error and the confidence threshold. As the confidence threshold increases, the available reference nodes decrease gradually. For SLMP, the dominant localization method is trilateration when the confidence threshold is below the turning point and then turns to self-estimation after the confidence threshold is above the turning point. Hence, the localization error of SLMP first decreases gradually and then increases rapidly. In comparison, STVF mainly selects the reference nodes according to the confidence values of their measurements. The decrease of the available reference nodes limits the selectable measurements and simplifies the confidence value of the measurements to that of the reference nodes. Therefore, the localization error of STVF increases with the confidence threshold gradually. In practice, we can properly lower the confidence threshold to permit more localized nodes to be reference nodes and control the quality of the measurements with their confidence values.

#### 5.3.3. Impacts of the Standard Deviation of the Measurements

**Figure 9:**
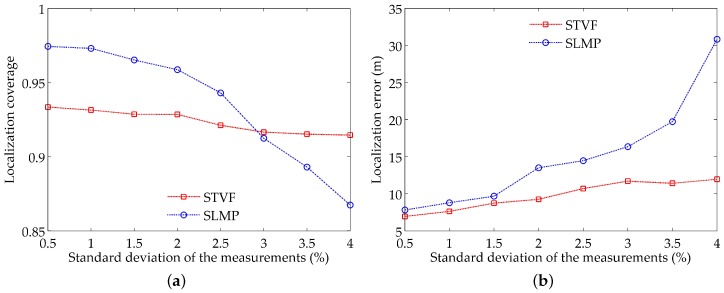
Impacts of the standard deviation of the measurements on: (**a**) localization coverage; (**b**) localization error.

In this set of simulations, we evaluate the impacts of the standard deviation of the measurements on localization performance. The values of the standard deviations are normalized to the real propagation delays. By keeping the parameter settings in Table 1, setting the threshold of the measurements’ number to be 2 and changing the standard deviation of the measurements from 0.5 percent to 4 percent, the localization results are illustrated in Figure 9.

From Figure 9a, we can see that the localization coverage of SLMP and STVF both decrease with the increase of the standard deviation of the measurements, and the localization coverage of SLMP falls more rapidly than that of STVF. There exists some critical value. Below this value, SLMP has better localization coverage, but, above this value, STVF outperforms SLMP. For example, as shown in Figure 9a, this critical value is 3% under current parameter settings.

Figure 9b shows us that the localization error of the two algorithms increases with the standard deviation of the measurements. Moreover, the localization error of SLMP rises faster than that of STVF. This is mainly because the trilateration method adopted in SLMP is susceptible to the measurement noise, whereas STVF can accommodate the noise using the proposed measurement model.

## 6. Conclusions

Focusing on the problems in UASN localization, including continuous movements of nodes, varying acoustic speed and dynamic noises, we have presented STVF, a novel localization method that fuses spatial correlation and temporal dependency information. In STVF, the mobility patterns of the nodes are characterized by the general state evolution model, and the variation of the acoustic speed and the dynamics of the measurement noises are captured by the measurement model. The states of the unknown nodes are augmented by regarding the expectation, the precision, the acoustic speed and the measurement noise as variables. The posterior probability distribution of the state variables of each unknown node can be optimized jointly using a variational filtering technique. The localized nodes with high confidence are then labeled as new reference nodes. We evaluate the impacts of the threshold of the measurements’ number and the number of the samples. The simulation results give the suggestions of selecting proper values for the two parameters. We also evaluate the localization coverage and accuracy of STVF and SLMP under different parameters, including the node density, the confidence threshold and the standard deviation of the measurements. The simulation results show that STVF reduces the localization error dramatically while maintaining approximate localization coverage with SLMP. Moreover, STVF is robust to the change of the parameter settings and works well even in sparse UASNs.

## Figures and Tables

**Table 1:** Simulation settings.

Parameters	Values
Localization area	500 m × 500 m
Simulation time	500 s
Localization period	1 s
Node number	200
The proportion of the beacon nodes	20%
The communication range of the nodes	80 m
The ratio of the standard deviation to the propagation time	0.02
$\bar{\Lambda }$	1/900I ${}^{†}$
$\bar{W}$	5**I**
$\bar{n}$	10
$\bar{v}$	1500 m/s
$\bar{V}$	1/100
$\bar{\alpha }$	3
$\bar{\beta }$	200
$N_{v}$	100
$N_{s}$	100
$\bar{\delta }$	0.9
$C_{1}$	0.7
$C_{2}$	0.2
$C_{3}$	0.1
ω	N(1,100)
$k_{1},k_{2}$	$N(\pi ,100/\pi^{2})$
ι	N(3,100/9)
$k_{3}$	$N(2\pi ,25/\pi^{2})$
$k_{4},k_{5}$	N(1,100)

${}^{†}$ The symbol **I** represents an identity matrix.
